# Choosing the best apple: counselling leaflets and technologies of communication in the history of reproduction

**DOI:** 10.1136/medhum-2023-012664

**Published:** 2023-07-04

**Authors:** Birgit Nemec

**Affiliations:** History of Medicine and Ethics in Medicine, Charité Universitätsmedizin Berlin, Berlin, Germany

**Keywords:** cultural history, Genetics, reproductive medicine, History

## Abstract

Historians have shown how the establishment of human genetic counselling in West Germany was characterised by several sociohistorical factors, in particular the impact of the legacies of Nazi biopolitics. These accounts have reconstructed continuities on an intellectual level which delayed a turn towards non-directive approaches, emphasising individual (emotional) well-being and voluntariness, and instead have prolonged a discourse that defined disability as an economic and social burden. However, while the distinct legacies of eugenics and racial hygienics are well researched, other factors that constituted counselling encounters, such as the ways of communicating reproduction and material objects’ roles in transformations of concepts, actors and their relations, have not been examined in detail. Drawing on the archives of a Marburg-based charity, this paper aimed to reconstruct these factors at the example of the production and circulation of a major family planning leaflet, *Our Child Shall Be Healthy*, developed ca 1977. In doing so, I want to suggest that connections between science, politics and economy were a key element in technologies of communicating reproduction. This essay approaches counselling as a communicative practice that was in continual productive engagement with different concepts of reproductive health. First, it argues that the communicative and paper technologies used in counselling interactions in West Germany changed in the aftermath of the worldwide thalidomide tragedy. Second, it argues that a novel approach to reproductive health emerged that focused on individual decision making as the basis of prosperity and emotional well-being. Taking a family planning leaflet as a site for reconstructing how people of different organisations, with different stakes and expertise converged in the design of a counselling encounter, this paper targets the crossroads of economic, political and scientific activities in the history of communicating reproductive health and reproductive risks.

## Introduction

**Figure 1:**
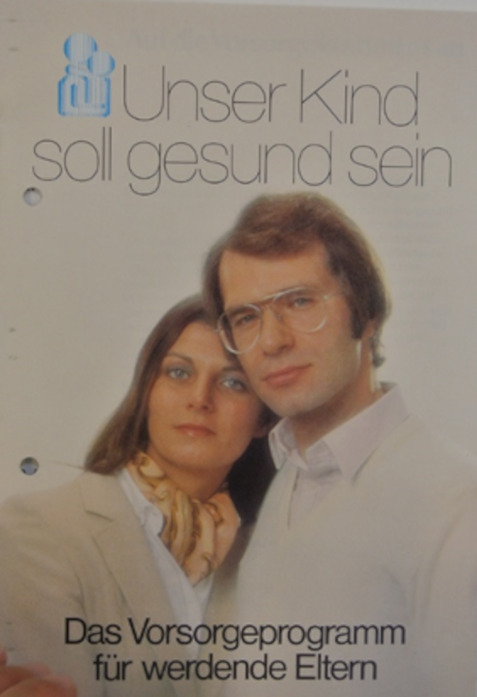
Cover of *Our Child Shall Be Healthy*. Foundation for the Disabled Child, n.d.

From 1977 to the late 1980s, the Marburg-based Foundation for the Disabled Child (FDC, Stiftung für das behinderte Kind) produced many richly illustrated, glossy family planning leaflets. Ranging between 80 000 and 130 000 copies per year, the 16-page leaflets contained written advice, images and checklists for the periods ‘before conception’, ‘during’ and ‘after the birth of the child’.1 They were entitled *Our Child Shall Be Healthy: Prevention Programme for Future Parents (Figure 1)*, which suggested that a standard hope of many people and families—that is, the good health of their child—would be fulfilled by ‘preventive’ acting . On the following pages, the foundation guided its readers through decision-making processes in so-called ‘family planning’.1 The leaflets were published in a decade of social transformation after the economic ‘boom’ (1970–1971, Doering-Manteuffel and Raphael 2008) and before the consolidation of disability rights movements. This was also a period of rapid technological and legal change in the field of reproduction and medicine. Following the introduction of amniocentesis in 1971 (Nemec and Zimmer 2019) and the liberalisation of abortion law in 1976, family planning, as the leaflet explained, ideally comprised one or more counselling encounters with medical experts who specialised in human genetics. It also comprised research into the counselled person’s family history, the interrogation of other family members (especially the mother and the partner’s mother), and the study of photographs and stories from family archives, as well as regular medical check-ups and, in some cases, prenatal genetic diagnosis and the termination of a pregnancy.1

The authors of the brochure presented as the norm a heterosexual, married, white middle-class couple, pictured full-page on the cover. With some historical distance, the couple looks pedestrian, bourgeoise, almost like a stock image of a late 1970s happy German couple. Without doubt, the images that the authors chose were intended to reflect wealth and social security, if not modernity. Moreover, the images captured strong relationships: they showed (in turn) relationships between two young adults; between parents and their baby; between an adult son and his mother; between an adult woman and her older, male doctor; and between a younger male doctor, a mother and a baby. The images emphasised the attachment and emotional and intellectual connectedness of the couple and their first, healthy child. By choosing the title, *Our Child Shall Be Healthy,* the authors took the position of the counselled couple and suggested a language that reflected confidence, self-determination and autonomy. As the leaflet contained not only texts and images but also full-page checklists, with spaces for readers’ notes and reactions, it prompted, at least to some degree, a two-sided way of communication between counsellors and counselled.

**Figure 2:**
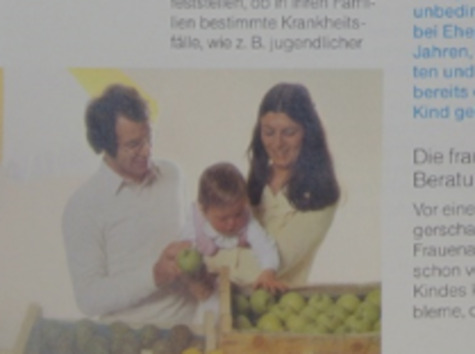
*Our Child Shall Be Healthy. Prevention Programme for Future Parents*. Archive of the Foundation for the Disabled Child, n.d.

In *Our Child Shall Be Healthy*, the couple’s emotional well-being was framed as the primary goal of a counselling interaction. Remarkably, this was implicitly linked to economic prosperity. In one picture (Figure 2), the authors illustrated their concept of reproductive decisions with capitalistic metaphors using the analogy of grocery shopping and the practice of choosing the best apple from a large basket. The foundation used the leaflet to explain principles of inheritance to their audience, using the example of the phenotype of fruit. Referring to free market economy and individual choice and benefits, they portrayed the nuclear family—parents together with their first, healthy, biological child2—as making reproductive decisions with confidence and as a strong moral and economic unit. As a powerful communication device that pertains directly to the meaning of ‘genetic counselling’, we can assume that the popular leaflet constituted many West German families’ first or only encounter with the agendas of clinical genetics.

Historians have recovered how, starting in the 1950s, scientists in West Germany at newly founded departments for human genetics referred to both established and newer concepts to make and communicate knowledge about reproductive health (Söhner and Krischel 2019; Schenk 2013; Thomaschke 2014; Nemec and Moser 2017; Cottebrune 2013). Following the thalidomide tragedy, in the mid-1960s, non-university or municipal, state and charitable institutions were established that aimed to offer counselling and funding for the prevention of birth defects. In 1972, the FDC opened the first genetic counselling centre in the central West German city of Marburg. The story of how the foundation’s chairman, geneticist Gerhard G Wendt, convinced politicians and audiences with his arguments to ‘prevent children with diseases’ to ‘minimise charges for our society’ (Wendt 1976) has become a fixture of our historical accounts of the continuity of eugenic reasoning in early reproductive counselling (Klee 2001, 272–3). ‘Most people who take care for the disabled are today in the situation of a man who desperately tries to scoop water from its house but who doesn’t think to clog the broken faucet’, stated Wendt, using a common public health metaphor, in an educational paper in a Christian newspaper to promote genetic diagnostics and counselling (Wendt 1976, 251). While acknowledging these intellectual continuities in science and medicine (Bösl 2009; Cottebrune 2015; Klee 2001; Petermann, Harper, and Doetz 2017; Schenk 2013; Söhner et al. 2022), which prolonged a discourse that defined disability as an economic and social burden, we still know little about other factors that constituted counselling encounters, such as the ways of communicating reproduction (Hopwood et al. 2015), and material objects’ roles in transformations of concepts, actors and their relations have not been examined in detail. This paper’s aim and innovative work are to reconstruct this package of social and material worlds (Clarke and Starr 2008), following the making and use of a counselling leaflet, as one among a set of technologies for communicating reproductive health.

Scholars in history of science and in the social sciences have pointed to the role of health activism and social movements in destabilising power relationships in medical encounters, and the production and communication of ‘counterknowledge‘ (Güttler et al. 2016) or alternative and non-expert knowledge, in the 1970s and 1980s, such as in feminist or patient-organised psychiatric encounters (Kersting 2003; Kline 2010; Morgan 2002; Raphael 2017). This has helped us understand the dynamic processes of knowledge and ignorance production in the history of medicine (Tuana 2006). However, focusing on critical and social movements and contrasting these with ‘expert’ discourses can obscure significant entanglements between the different actors and their modes of reproductive and sex counselling. Every form of communication in reproduction and its material cosmos—books and leaflets, photos, notes, transcripts and meeting protocols produced and used—unites different actors and funding sources. The same concepts could provide the material for a counselling leaflet for married couples, a student textbook, a critical radio show or a popular lottery. For the FDC, the family planning leaflet was a tool for communicating a specific concept of risk and prevention in a rather directive way to couples and families, that is, using ‘checklists’. It was also a tool for advertising counselling interactions in clinics, private practices, schools and other public spaces where more doctors, teachers and people would encounter these concepts and related practices. Also, the leaflets were directly sent to people’s homes, especially to low-income households. Today, following the FDC’s movement from Marburg to Frankfurt to Berlin, counselling is offered at a downtown campus of the city’s major university hospital, the Charité.

The design of a leaflet to be used in reproductive health counselling is a process that involves several actors and concepts of ‘family’, ‘risks’ or ‘prevention’ (Aronowitz 2015; Löwy 2018; Schlich and Tröhler 2006). This makes reproductive counselling leaflets an important site for investigating practices of communication and decision making in the history of reproduction (Andrew 2016; Hopwood et al. 2015). This paper reconstructs the institutions, experts, stakes and social contexts that constituted genetic and reproductive health counselling in post-thalidomide West Germany. I mainly draw on archival and published evidence from FDC. For several decades, the FDC’s counselling services have been a place where healthcare professionals help parents and patients interpret the results of genetic tests and negotiate decisions about reproduction and treatment. Today, a cornerstone of Berlin’s infrastructure in reproductive healthcare, the FDC has a 70-year history in the provision of services and knowledge that matter to the experience of (West) German patients and families. I will focus on the largely unwritten FDC early history and use counselling leaflets as an entry point to reconstruct encounters between couples and FDC counsellors, who were often trained in human genetics and in talking about reproductive health. This conversation was two-sided, but, drawing mainly on FDC archival materials, my approach centred on the counsellors’ side. From there, I expand my view to decision-making processes and emotions in the social and material cosmos of communicating reproductive health. The aspects that I reconstruct range from the establishment of infrastructures, the targeting of audiences and the positioning within a whole range of counselling approaches to the making of a brochure and its transformation, with the reactions of audiences and other institutions. In so doing, I make two inter-related arguments. First, I argue that communication in counselling interactions in West Germany changed in the aftermath of thalidomide and related transformations of funding and public as well as political debate on birth defects. Second, I argue that a novel approach to understanding emotionally laden decision making in reproductive health emerged in this period in West German history that was shaped by institutions such as the FDC and its range of stakes and entanglements between science, (pharma)industry and politics. It focused on individual choices securing prosperity. Moreover, audiences learnt that ‘right’ choices were the basis of emotional and economic well-being of individuals and families. Only with the stronger formation of disability and feminist advocacy, these entanglements and arguments, in particular the underlying economic argument, were challenged (Bradisch, Feyerabend, and Winkler 1989; Sierck and Radtke 1989).

## Technologies of reproductive health counselling before the 1970s

From 1920, reproductive health counselling was established as a key aspect of social medicine in the Weimar welfare state (Weindling 1998). Publicly funded institutions, such as municipal marriage counselling centres, provided the space for encounters between doctors, often in a double role as health politicians of ‘welfare eugenics’, and couples and women. Pregnant women were often seen as mainly responsible for the health and development of the fetus. Some researchers also conceptualised fetal development as open to external manipulation (Buklijas 2014; Helduser and Dohm 2018; Richardson 2021), such as maternal ‘impressions’ during pregnancy. Materials, most of all illustrated wallcharts, exhibition posters and educational films, were used to communicate a utilitarian, hierarchical and directive approach to communicating reproduction, often focusing on the risk of sexually transmitted diseases.

From 1933, in contrast to the voluntary nature of counselling in Weimar, the national socialist state subordinated the interests of individuals entirely to a fostering of the ‘Volkskörper’ and laws and public health departments were installed to control individual reproductive decisions, from prohibitions of marriage to forced sterilisations (Ley 2003; Westermann 2009). The images and charts used in counselling encounters communicated the assessment of a couple’s ‘eugenic fitness’ based on physical appearance and family history, as well as the stigmatisation of factors that were expected to make the offspring dependent on medical assistance or social benefits (Matz 1980).

After 1945, physicians, medical specialists, public health officials and medical researchers found themselves in a judicial vacuum in terms of the legitimacy of interventions in reproduction. Structural, legal, personal, and intellectual continuities and hybridisations with Nazi racial hygiene went hand in hand with a reorganisation of the national healthcare system, with the governance in the hands of the federal states. This decentralisation and larger transformation also applied to communicating reproductive health. Ecclesiastical institutions, alongside public health departments, installed counselling and information centres funded by health insurance authorities, while also geneticists, compared with the Anglo-American world, slowly established counselling units and related communication technologies (Cottebrune 2015; Schenk 2013; Thomaschke 2014; Waldschmidt 1996).

What factors explain the new approach to communication in reproductive counselling, by the late 1970s, focusing on individual choice as the basis for happiness and satisfaction and the framing of economic decisions as analogous to reproductive decisions? From the 1960s, a vast new field of counselling activity developed in West Germany. This was linked to the establishment of several associations and foundations in the field of reproductive counselling during the 1950s, such as the German Society for Marriage and Family (1952), later Pro Familia. However, the major factor was the emergence of medical and moral support structures for children and families after Germany became the starting place of the thalidomide disaster of 1960/1961, when thousands of children worldwide were born with severe limb malformations or died young because of the then unknown teratogenic effect of intake of a drug during early pregnancy. The drug was produced by the German enterprise Grünenthal, and the tragedy was exacerbated in the FRG by high consumption of pharmaceuticals, lack of information about adverse effects, and absence of effective drug laws or regulatory oversight (Daemmrich 2002; Kessel 2017; Kirk 1999). Examples of these support structures are the Campaign Sorrow Child (1964), the Heidelberg Rehabilitation Trust (1966) and the FDC (1967). These charities through campaigns stimulated a public debate on reproduction, disability, religion and politics (Herzog 2018). Bringing together members from different societal realms, they also served as platforms of exchange between political, scientific and industrial institutions, experts and contexts. Moreover, by the publication of leaflets, besides books, journals and TV spots, in parallel with the development of a counselling infrastructure, new concepts of risks and decision making were introduced. A whole material cosmos encountering people at their homes, by mail or broadcast played a major role in transformations in the history of counselling in West Germany’s postwar decades. In the following sections, my analysis will focus on the production and dissemination of one of the most popular counselling leaflets in West Germany, named *Our Child Shall Be Healthy*, edited by the FDC from ca 1977 under the supervision of Gerhard Wendt.

## Marburg model

The University of Marburg was one of the few places where genetic counselling was offered more systematically prior to the late 1970s, but as a non-university, charity-run enterprise. Gerhard G Wendt, a student of Hans Nachtsheim, eminent Berlin zoologist and geneticist, who specialised in the experimental study of hereditary diseases, introduced the concept from the late 1960s (Klee 2001, 274). Wendt studied medicine in Rostock, Würzburg, Berlin, and Prague during the war, worked in the Air Force and as sanitary officer, as well as in Bethel, a church-run psychiatric hospital. The hospital refused to participate in the centrally planned Nazi mass murders of people in hospitals (‘Aktion T4’), but still, patients with low chances of integration into the labour market were often considered a financial charge for the public health system. Wendt took some aspects of this reasoning into his postwar career. After a year at Münster’s legal medicine and pathology department and finishing his *Habilitation* in anatomy (1952), visiting fellowships at Hans Nachtsheim’s newly founded department for human genetics at Free University Berlin and with geneticist and eugenicist Tage Kemp in Copenhagen, brought him into human genetics. In the aftermath of thalidomide, he investigated ‘genetic defects caused by civilization’ (ie, caused by radiation) and its ‘social management’ (Wendt 1962). After being appointed professor for human genetics in Marburg (1963), he launched a publicity campaign for genetic counselling. Referring to a 1969 WHO report on genetic counselling, he organised a symposium including scientists, politicians and journalists (‘Genetics and Society’ 1969; Wendt and Baitsch 1970; Schloot 1984, 9). Together with the availability of amniocentesis, this report paved the way for a pilot project for genetic counselling in 1971 (Bundesministerium für Jugend and Familie und Gesundheit 1979, 7; Nemec and Zimmer 2019).

Wendt established the first counselling centre in Marburg in 1972 as part of the pilot project. Funded by the Federal Ministry and the Volkswagen Foundation, he appointed a team of six clinicians with different specialisations (mostly gynaecologists and paediatricians) to build a unit for ‘genetic family planning’ (Wendt 1973, 352; [Bibr R133], 27). University facilities were scarce, but Wendt activated his non-university contacts. The FDC had just opened a genetic counselling centre in Marburg and left a whole floor to Wendt’s ‘genetic hour’ (Svejcar 1979, 97). After a period of counselling on request, between 1972 and 1974, a cohort of 2844 people was counselled within a more systematical framework (Wendt 1979, 24). This was in collaboration with biologists and biochemists culturing and analysing chromosomes for genetic tests. Wendt described how counselling worked in practices as follows: if a person seeking advice or who had been referred by a physician visited the unit, counsellors first tried to contact as many family members as possible. Then they arranged meetings with the members of what they termed ‘counselling family’ (Wendt 1979, 15). This included personal meetings as well as collecting documentation, that is, photographic documentation of the counselled, or picking them up, by car, from their homes, carrying them to a specialist and exchanging patient data with specialists. The counselling meetings took place in the counselling unit, and the goal was to identify diseases or anomalies that were considered ‘counselling relevant’ (ie, to run in families). This process was based on a pedigree or genetic test. As the last step, ‘risk numbers’ for reproduction were calculated and communicated by telephone, in a personal encounter or by mail (Wendt 1979, 12).

The popular weekly newspaper *Die Zeit*, targeting middle-class audiences, regarded the Marburg pilot project as an ‘ideal trial’ (Löbsack 1975). Indeed, as historical accounts have noted, the Marburg unit became a model for institutionalised counselling in West Germany, from clinics and private practices to departments for human genetics and non-university institutes. In 1977, counselling was offered in 37 centres; by 1982, this was 41 (Stengel-Rutkowski and Murken 1973–1982 [Bibr R66]). In these early years, some places reported a quadruplication of counselling cases (Koch and Schwanitz 1977; Tünte 1979, 76–77). By 1975, Wendt’s FDC-run working group had grown into a polyclinic with a staff of 10 at the University of Marburg ([Bibr R134], 32). In 1981, over 30 ‘more or less efficient’, in terms of counselling cases per year, counselling units had been established, as reported a conservative, probusiness weekly newspaper ([Bibr R133], 27). As part of a larger development, that is, the reintegration of human genetics in medical education (licensure act; 2-year specialisation for doctor of medicine (MD), 1973), health politicians called Wendt’s Marburg model the ‘pacemaker’ for the journey from the trauma of Nazi eugenics to ‘new opportunities deriving from genetic diagnostics and counselling’ (Bundesministerium für Jugend and Familie und Gesundheit 1979, 7).

In this initial period, counselling was conceptualised as having two objectives: as the provision of ‘help in decision making in family planning’ and to ‘prevent’ and ‘reduce […] genetic diseases’ (Bundesministerium für Jugend and Familie und Gesundheit 1979, 46) that were regarded a ‘sorrow’ (Bundesministerium für Jugend and Familie und Gesundheit 1979, 7). Many scientists considered their alleged increase an ‘urgent task’ (ibid.; Wendt 1973, 351). Wendt’s favourite concept of reproductive health blended an older concept of degeneration (by steady accumulation of genetic ‘load’) with a rhetorical delineation from coercive, state-enforced Nazi eugenic practices, with newer approaches. The result was a strong union of genetic testing and rather top-down, directive approaches to counselling to support lower-class and middle-class heterosexual couples to get healthy offspring (Wendt 1973; Wendt 1974; Wendt 1976; Wendt and Theile 1974). In short, Wendt was known for an understanding of reproductive health counselling as ‘primary prevention’ of disability.

To this end, the collaboration between Wendt and the FDC to create the first counselling unit in Marburg relied on the making of a family planning leaflet. A six-page leaflet entitled *Genetic Counselling Helps Us*…, the predecessor of *Our Child Shall Be Healthy*, was produced in collaboration with a local working group for health education. The first edition in 1973 (the year of the oil crisis) had a print run of 1 million copies. Designed to reach audiences beyond the pilot unit, in adult education centres, women’s clubs and parents’ organisations, its main aim was to encourage readers to start a conversation with their trusted doctors about ‘hereditary health’, their family and ‘potential risks’ (Wendt 1973, 397). In this leaflet, as well as in other formats of educational material or events, organised in the district surrounding Marburg, Wendt gave directions on how to ‘plan one’s reproductive behaviour’, which had to start before conception (Wendt 1973, 397; Wendt 1974, 123). However, in parallel with this emphasis on personal freedom in decision making, bearing children with certain genetic traits was defined as undesired for economic reasons. ‘We cannot afford anymore to just care for the disabled people that exist today. We cannot afford it in the interest of the people who are ill and not in the interest of the whole society’, Wendt stated in a TV interview to promote genetic counselling (Wendt, n.d., quoted from w.a 2017, min 01:50–02:08). Accordingly, the communicative strategy was to define disability as a risk that couples should not ‘dare to’ take, with people with disabilities as a cost factor, and to link disability prevention to emotional well-being while leaving decision making to individuals.

Similar to what many migrant workers experienced after the boom and the end of the recruitment agreements between Germany and Turkey and other South European countries (in force 1955–1973), the leaflet was used to communicate that the time when ‘Everybody was needed’ (Eisenbichler 2011) was over. This strategy would be repeated with Wendt’s other collaborations, including the making of *Our Child shall be Healthy* with the FDC.

## New leaflet following audience evaluations

A leaflet used in counselling encounters is a technology that prompted, at least to some degree, a two-sided way of communication between counsellors and counselled, including the testing and refinement of communication practices according to audience reactions. Despite the rapid growth of counselling centres in in West Germany and West Berlin, by 1970, published material to be used in reproductive health counselling was rare. Friedrich Vogel and Walter Fuhrmann’s *Genetic Family Counselling: A Guideline for Students and Doctors* (1968)3 was the first major book on genetic counselling in German and had been a success; it was both translated into English and re-edited (Fuhrmann and Vogel 1975, Fuhrmann and Vogel 1982). However, its main audience was medical experts, and its principal task was to reintroduce genetic counselling as a subject to medical students (Fuhrmann and Vogel 1968, V). In doing so, the *Guideline* explained basic principles in heredity and listed information on a number of medical conditions. Only in a short chapter, ‘The final consultation’ (in a later version revised and expanded to ‘Psychological and social aspects’), the structure of the counselling situation, the emotions and empathy it triggered, as well as the communicative dynamics were reflected briefly as aspects of counselling situations. In the following years, communicative technologies diversified, as well as audiences.

The audiences who actively approached counselling units, as was the case with general medical advice, were limited. Counselling within the Marburg model was free of charge. To reach individuals from lower social background, it was even accessible without a letter of referral, unlike other areas of the German healthcare system (w.a 2017). After the first phase, the Marburg model was evaluated. The evaluative study had revealed a difficulty to reach people in Marburg’s periphery and those considered to be ‘from a lower class’. Another factor pointed to the communication practices within counselling encounters. As was revealed by the evaluative study, 25% of patients had made their reproductive decisions against the advice of the counsellors; 50% had reported the counselling session as ‘not helpful’, and a large number who described the counselling session as ‘helpful’ had not remembered the advice given correctly (Mahn 1979, 91). The format of the advice, it occurred to Wendt, was eventually a major factor. Many patients had apparently reported that they were unsatisfied with the communication practices. They had difficulties to understand the oral communication of risk numbers and, even more, to put them in relation to taking reproductive decisions. Many would have preferred a more directive advice in counselling encounters (Wendt 1979, 12). This was when Wendt started to raise funding for the making of a new counselling leaflet.

In a first step to broaden audience and multiply encounters, from 1973 to 1974, *Genetic Counselling Helps Us…* was sent specifically to families who were considered in need of social services (Wendt 1974, 125). In a paperback on genetic counselling for publishing house Herder’s popular political series ‘Social Problems’, Wendt explained what was meant by this broad category: households with adults who were classified to have ‘basic jobs, under-average intelligence and low income’ (Wendt 1974, 125–6). We cannot trace in the sources available how these families were defined, identified, reached, or how and if the counselling situations continued, after receiving the leaflet. However, when this campaign proved unsuccessful, Wendt decided to design a brand-new leaflet, embedded into an education initiative in public schools. His reasoning was ‘School leavers, should know hereditary and chromosomal diseases, the person should know that diseased offspring is a burden to families and a danger for the hereditary health’ (Wendt 1974, 125–6). In addition, counselling materials would be used in trainings for MDs, as well as for teachers, especially in biology and social studies. Other points of dissemination would be mass media campaigns in local and nationwide TV; ideally, an evening series on ‘hereditary diseases’ (Wendt 1974, 130); advertisements for the counselling unit in daily newspapers, magazines and in well-established medical mailings sent to people’s homes, such as the letter of the *German Employees’ Health Insurance* (*DAK*). To support communicative practices, Wendt activated his network. In the following year (1975), he signed a contract with a professional public relations (PR) company, on behalf of the FDC, to develop *Our Child Shall Be Healthy*.

## Transformations in communication practices after thalidomide

Major changes in patterns of funding, communication and expertise help to explain the making and content of *Our Child Shall Be Healthy*. After thalidomide, new civil societal actors had appeared on the scene. From the mid-1960s, welfare unions, self-help initiatives, charitable organisations and federal initiatives competed on a newly established market for research funding, political lobbying and public influence. Well into the 1970s, their communication activities were shaped by mutual demarcation processes and the division of the field.4 As Sabine Schleiermacher showed for the case of *Pro Familia*, charity representatives active in reproductive health counselling managed to form a particular expertise within the federal republican social state and participated, by lobbying, in sociopolitiocal policy making ([Bibr R137]referring to Raphael 1998, 232). Charities affected the politics of research funding (DFG 1966, 23). Moreover, as historian Dagmar Herzog argued, notions of sexuality and reproductive health in West German society were sustained by these organisations (Herzog 2018). The FDC, founded in December 1966 in Bonn, was one of them.5

In 1966, approximately 4–5000 children born in Germany with severe limb malformations due to thalidomide turned 6 years old. As we can see in archival correspondence, their school enrolment was a watershed in debates about reproductive health. It revealed a lack of societal and state support structures for children with special needs, and it triggered debates about the risks of disability to productivity and economic growth. In January 1966, Ludwig Hamm, solicitor and politician for the economic–liberal Free Democratic Party (Freie Demokratische Partei, FDP) brought the idea of a national ‘foundation for the support of physically handicapped children’ into the German Bundestag. Notably, Hamm had chaired the Bundestag’s health committee in the immediate aftermath of thalidomide. ‘In the face of the upcoming school enrolment of the children with malformations due to Contergan, rapid acting is necessary’, he stated, now state secretary for economy and transportation. However, ‘this task’, Hamm argued, ‘was too big for state welfare or welfare unions’, and a union of science, industry, workers’ union, church and other societal spheres was needed. He suggested to establish a foundation, at the example of the US March of Dimes, a charitable organisation that specialised on the prevention and disability. High-ranking members recruited from politics, industry, welfare unions and science, in particular human genetics, gynaecology and paediatrics, would guarantee its success. In the following summer (1967), the FDC’s founding meeting was held in Bad Godesberg, a district of the Republic’s capital Bonn (Nitsch 1974, 193).

Meanwhile, audiences in the Federal Republic, after thalidomide, had encountered a variety of material on reproductive health. With the *Campaign Sorrow Child (Aktion Sorgenkind),* they learnt about children damaged by thalidomide in a TV quiz show and lottery with popular moderator Peter Frankenfeld (from October 1964). Millions of viewers followed the children’s fate and hardship, as it was hoped this would raise funding. To this end, the children were filmed during play, portrayed as ambitious and brisk, but also depressed and lonely (BArchKreference). The parents and other adults did not exist in the narrative. Moreover, another charity, *Foundation Dolphin*, shocked families and divided doctors over the question of outreach strategies, with images of severely disabled children.6 The Heidelberg Rehabilitation Trust, focused on social rehabilitation, raised public awareness for the special infrastructure needed for children with physical impairments, such as in schools and workplaces. With its funding of a counselling unit at the University of Heidelberg, it set new standards on the use of scientific images in counselling settings (Osten 2012, 165).

Against this backdrop of ‘thick network’ (Herder-Dorneich 1994, 563) of charities, institutions and foundations focusing on disability, birth defects and reproductive health, the FDC changed its profile.7 ‘Solidarity with the disabled children is something everyone has to train everyday’, health minister Käte Strobel had stated at the FDC’s opening symposium.8 However, from the early 1970s, with a new family planning leaflet, *Our Child Shall Be Healthy*, to stake out its position among the competitors, the focus was moved from acceptance and assistance9 to primary prevention of birth defects10 (Nitsch 1974, 193, 195).

## Leaflet as a paper technology of counselling encounters

Producing a counselling leaflet of decent quality and quantity is a considerable investment with high return prospect for a counselling institution, charity or geneticist. However especially from the 1960s, with a developing market and competition for research funding, political lobbying and public influence, reproductive health counsellors turned to advertising agencies for effective public outreach. With the end of the pilot project and the question of follow-up financing for the genetic counselling unit (Bundesministerium für Jugend and Familie und Gesundheit 1979, 7), the FDC’s advisory board set on a ‘PR-offensive’,11 with the design of a new counselling leaflet, as the core of its ‘prevention programme’.12 How did the design and *paper technology* (Hess and Mendelsohn 2013) of a counselling leaflet, its material and non-material features, give counselling encounters a specific form?

In early 1975, the FDC signed a contract with the advertising agency Gabler, a Stuttgart-based, family-run business.13 The advisory board decided to invest in a new leaflet, estimated at 1800 DM (first edition/1000 copies),14 and directed to lay audiences. Gabler was entrusted with the design, Georg G Wendt with the content.15 We cannot trace in the sources available how the design process developed in detail. It is mentioned that ‘earlier material’, very likely the leaflet *Genetic Counselling Helps Us*, was not only used as a model but also considered ‘outdated’ and discarded in the foundation’s administrative office in Frankfurt’s Kennedyallee,16 or sent to pharmacies to be used up.17 Several months later, the first draft of *Our Child Shall Be Healthy* was presented. It was double the volume of its predecessor: a 16-page glossy leaflet, in a white-and-blue colour scheme, with longer text parts, checklists and rich illustrations. A heterosexual, healthy couple in its early 30s was the main actor. Assumingly, thereby responding to the results of the audience evaluation, the leaflet gave counselling encounters, and the different actors and practices involved a much more straightforward form: combining the a specific vocabulary and medical reasoning with directive expert advice, with a focus on individual choices and decision making as the key to prosperity and emotional well-being.

## From the choice of the best apple to the conscious choice of child

*Our Child Shall Be Healthy* marked a new direction in the communication of reproductive health. The cover of a version from ca 1979 shows a heterosexual, married, white, well-situated, attractive couple in their early 30s (Figure 1). A woman, on the left, and a man, on the right, are looking directly into the camera; they are captured slightly from below and their viewing directions are slightly crossed. The counselled do not appear insecure or worried, after a paternalistic, directive or even oppressing counselling situation that the parent generation of young adults in this decade might have encountered. They seem confident. They represent a strong unit, happiness, satisfaction, security and eagerness. The brunette woman wears a beige blazer and a silk scarf, knotted in a staid manner; the man, brunette with a receding hairline, wears a white shirt, top button open and aviator goggles. The pedestrian, bourgeois, stock image-like portrait of a happy German couple is very different from visual counselling materials of the 1930s and 1940s. We do not see any anti-Semitic references, ‘Aryan’ stereotypes, working-class couples, chronic illnesses or stigmatising references to a certain sexual behaviour or to disability. Moreover, neither do we see any other reference to age, race and class, often addressed by educational initiatives since the first counselling units opened in Weimar (Sauerteig 1999; Sauerteig 2001). However, as argued for other cases (see paper in this special issue on the Czech Republic), we can assume that the authors were well aware that this image also communicated with those who considered themselves and who were considered outside that white, middle-class, bourgeoise category, like working class audiences or immigrant workers.

Historiographies of sex education in West Germany have noted a shift from ‘shame’ to ‘empathy’ (Laukötter 2021), following the 1960s shift to a more liberal public discourse and depiction of sex in the country’s highly conservative society (Schwarz 2009). With its leaflet, the FDC targeted a conservative middle-class, which it guided through decision-making processes in family planning. The counselling was considered to convey empathy and react to individual needs, as well as communicate clear directions in individual and public health. In the first section, titled ‘Prevention Matters’, readers were introduced to the topic through the example of a medical case study. This was the standard approach not only in medical journals but also in other counselling materials, such as in Vogel and Fuhrmann’s guideline (Fuhrmann and Vogel 1968).

In an encounter with a medical specialist, the leaflet informed that the woman had learnt of a presumed disability of her child. Readers, unsettled by the fact that the woman had assumed her pregnancy as ‘completely normal’, learnt that such cases could be ‘prevented’ (FDC n.d., 3). In the second section, ‘Family Planning’, readers were given a picture of the couple they would recognise from the cover, now with a toddler in the arms (FDC n.d., 4). In the following paragraphs, decision making in family planning was explained using the example of grocery shopping and an attention for economic principles. The family, or more precisely, the first, healthy child chooses the next apple from a large basket. The child is in the centre of the parents’ attention and, by the composition of the image, placed in the vanishing point of their lines of sight. For readers, the analogy, transported by text and image, was clear, from the choice of the best apple to the conscious choice of a child.

Principles of free market economy, individual decision making and profit were presented as key elements in reproductive decisions. In addition, parent–child relationships were conceptualised, in a neo-liberal sense, as a result of beneficial individual choices and decision-making processes. Not only the West German couple, the image argued, but also the nuclear family, as a strong moral unit, followed the FDC’s lead and took reproductive decisions with confidence.

## Communication of individual decision making as securing financial and emotional well-being

Class, as in other educatory projects, was a central category. The couple pictured in *Our Child Shall Be Healthy* is from a higher-income bracket; she, maybe a teacher, likely with an academic degree; he, maybe an engineer in one of the country’s leading industries, automotive or chemical—the sectors that stood for West Germany’s regeneration and economic boom. Readers would assume that the couple lived in a wealthy, conservative, rather bourgeois, mid-size city in the south, in one of the regions that would become net contributors after Germany’s reunification. Historians of advertisement and consumption have argued that with the oil crisis (1973), cars, as major ‘carriers of cultural and social processes of self-positioning’, were advertised to West Germans in a ‘rational emotionality’ (Neumaier 2017, 543), being a mix of emotional arguments (‘fun’, ‘happiness’, ‘adventure’, ‘safety’ and ‘modernity’) and rational decision making (‘economical’ and ‘efficient’). With the second energy crisis of the late 1970s, economic arguments and individual (social) resources dominated the advertisement material, more so than larger ecological responsibilities (ibid.). Couples that considered themselves as belonging to this new ‘rational’ middle class, who decided Volkswagen’s new compact car Golf, for example, to secure ‘individual mobility’ (ibid.), were also the target group of *Our Child Shall Be Healthy*. However, behind rational arguments, the leaflet also communicated emotional messages.

Which emotions and viewing experiences of West German audiences were evoked in *Our Child Shall Be Healthy*? Young adults, socialised in the German healthcare system after 1945 and reaching child-bearing age in West Germany around 1970, encountered decision making in a context different from the more collectivist approach in East Germany. Witnessing a period of economic meltdown and looming financial crisis, after the economic boom (Doering-Manteuffel and Raphael 2008), when full employment ended, their reproductive decisions, they learnt from the FDC’s Prevention Programme for Future Parents, were also expected to be financially beneficial decisions. These communicative strategies were grounded in older explanatory standards (disability framed as a financial burden) and the new approach of an individualisation of (economic) risks (Ewald 1993; Aronowitz 2015; Löwy 2018). In doing so, *Our Child Shall Be Healthy* also linked reproductive decisions to the protection of the hard-won prosperity of postwar West Germans.

**Figure 3:**
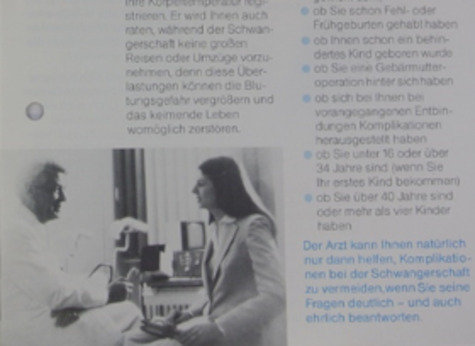
Counselling as a cultivation of intimacy. Foundation for the Disabled Child, n.d., 3.

Communication in counselling, by the leaflet, obtained the form of a cultivation of intimacy, trust and safety, that is, between a young and prosperous middle-class couple and a male, upper class doctor (see Figure 3). This intimacy implied a certain directiveness, as we learn from the aim of the encounter on the side of the counsellors, to ‘foster responsible parenthood’ (Wendt 1970). It implied counselling as an asymmetrical relationship between experts and non-experts. ‘Naturally, the doctor can help you only to prevent complications during pregnancy if you answer his questions frankly’, readers of the leaflet were informed (FDC, n.d., 3), thereby devaluing a potential wish of the counselled to keep certain information for themselves. In the leaflet, individual responsibility and yet also trust in medical authority were foregrounded as the emotional cornerstones of what was termed prevention (ibid.). This development was not confined to reproductive health but can be interpreted in the framework of what scholars described as the advent of the concept of a ‘counselled self’ and its repertoire of technologies of the self, as a crisis management strategy in the long 1970s ([Bibr R139]). Only retrospectively, studies would reveal that this form of communication raised the worries and fears of many parents (Nippert 1984).

## Later career of *Our Child Shall Be Healthy*

The first edition of the *Our Child Shall Be Healthy* met with a positive response and the demand increased.18 In this regard, some board members suggested to refinance the production, now calculated with 10 000 copies, with advertisements, a decision that was renewed in the following year, after the first edition proved ‘highly successful’, as Wendt reported to the advisory board.19 With 300 requests per day, delivery was behind schedule and he hired an assistant.20 By 1977, the first edition (100 000 copies) was sold out. To meet the orders, Wendt organised a reprint (2000 copies).21 In 1978, the first edition reached 120 000 copies. Two-thirds had been requested by prospective clients; one-third was disseminated over pharmacies, as an inlay in the popular and free-of-charge *Journal of Pharmacies*.22 Wendt successfully applied for additional funding with the Ministry of Education and, together with paediatrician Kurt Hartung, produced a revised version of the counselling leaflet.23

Revisions cannot be traced in the archives in full detail. However, from 1978, they were a key topic at the FDC meetings, now held at Wendt’s Department for Human Genetics at Marburg’s Robert-Koch-Straße 7, a historical building in the university district. Also, minutes of these meetings included exchange with major West German institutions in medical self-administration (ie, the General Practitioners Association, the Federal Doctor’s Chamber, the Bavarian Doctor’s Chamber, the Federal Union of German Public Insurance, the Union of private Insurance, and the Associations of Gynaecologists and of Paediatricians). In turn, with this stronger support network, the leaflet circulated among broader audiences in screenings and preventive check-ups in general practices24; patients received it from specialists they trusted, in gynaecological and paediatric practices25; women encountered it at maternity and infant welfare services; and pupils and teachers encountered it in schools.26 Remarkably, in the district of Hesse, it was handed over to couples by the marriage registrar ‘to emphasise its meaning’ and was displayed at the registry’s office.27 Moreover, it contributed to the content of popular TV shows, as *Campaign Sorrow Child* (ZDF) and *Campaign Prevention* adopted its content.28 In other words, the *paper technology* of the counselling leaflet gave counselling encounters a specific form in a variety of contexts.

As audiences and dissemination of the leaflet had reached a new peak, by 1979, Wendt resigned for health reasons from his FDC duties.29 However, the career of *Our Child Shall Be Healthy* did not end here. From the early 1980s, with new funding sources, including the *Federal Centre for Health Education* (BZgA),30 demand remained high31 and a re-edition followed (1986).32 Just before the fall of the Berlin Wall, an order of the Federal Union of German Pharmacies led to a reprint of 380 000 copies of *Our Child Shall Be Healthy*,33 guaranteeing that also in a unified Germany, the leaflet structured women’s, families’ and experts’ ways of attributing meaning to family planning, thereby setting standards in the communication of reproductive health.

## Civil societal networks behind a technology of counselling and health communication

Every counselling interaction and its visual and material world emerge from the collaboration of different actors and funding sources. Historians have pointed to the role of civil societal participation and the rise of organisations at the intersections of science, politics and industry in German history, as well as to a change in the relation among medicine and public or societal discourses in the postwar decades (Schleiermacher 1989; Stöckel 2014). From the 1950s, the close ‘entanglement’ they formed to push through lobbying interests led to a change from party democracy to organisation democracy (Herder-Dorneich 1994, 563). Reproduction was one site of intense scientific, medical, social, political and ethical change, and, as such, a crossroads for rich traffic between the biological, medical and social sciences, medicine, industry and politics (Schleiermacher 2004).

Even if geneticist Georg G Wendt for a long time was the moving force behind *Our Child Shall Be Healthy*, the leaflet was a collaborative project. The FDC was a consortium of powerful, primarily male politicians, industrialists and scientists, with the federal president initially planned to act as its patron. Members included Curt Engelhorn (co-owner of the pharma giant Boehringer Mannheim) and Hans Harms (president of Merck A.G. Darmstadt), Paul Lücke (minister of the Interior), Elisabeth Schwarzhaupt (minister of Health) and Gerhard Stoltenberg (minister of Research) among government representatives; Ludwig Hamm (FDP/ economic-liberal party) among the political delegates; and representatives from the social partners, such as president of the employer’s union Hanns-Martin Schleyer (later kidnaped and murdered by the RAF).34 Members included top-ranking scientists, such as paediatricians Kurt Hartung and Kurt Nitsch, or geneticists, Karl-Heinz Degenhardt, Horst Bickel, and Jan Murken. From 1977 to 1984, the counselling leaflet was FDC’s most important publication. Moreover, FDC members were active in the design, the financing and the dissemination, and the placement of the leaflet, communicating a specific concept of prevention and decision making in reproduction in private and public arenas.

Protocols of the board meetings and annual balances show the different levels at which the production and use of a counselling leaflet is shaped by alliances and collaborative relationships between different professional groups and their agendas. Amidst the thick network of powerful post-thalidomide charities, audiences, funding sources and channels to disseminate *Our Child Shall Be Healthy* were at stake. This was especially after access to FDC’s major source of funding, equalisation and compensation payments released by Contergan/thalidomide manufacturer Grünenthal, was cut off. In 1972, the National Foundation for the Disabled Child was established to administer its distribution, taking the place of smaller charities.35 This was when FDC members stepped in. Using their contacts, such as to the Federal Doctors Chamber (Ärztekammer), they launched massive publicity and fund-raising initiatives. FDC members, who were also members of the Green Cross, a major postwar welfare union with a strong network and marketing department, guaranteed support in PR and mass media strategies (Nitsch 1974, 196). One major step was to place the leaflet as an inlay to the apothecary’s magazine (*Apothekerzeitung*) and the doctors’ journal (*Ärzte-Zeitung*), thereby guaranteeing a dissemination to vast West German audiences.36 Other members ‘acted on’ circuit judges, so that FDC would profit from compensation payments (ie, 1974: 1490 DM, 1975: 1600 DM).37 Finally, the headquarter, at Frankfurt’s central Kennedyallee, a 57 m^2^, two-room apartment, was left to FDC by pharma giant Farbwerke Hoechst AG for a symbolic rent only (186 DM).38

The leaflet as a technology to frame reproductive decisions, by the late 1970s, as analogous to economic decisions, reflects significant entanglements between the different FDC members and their concepts of reproductive health. One example is the integration of the concept of ‘degeneration’ into the counselling leaflet. Under the impression of atomic armament and thalidomide, Wendt, just like other researchers, predicted a rise of ‘mutagenicity’ in ‘future generations’, parallel with a decline in ‘natural selection’ due to support structures and the healthcare system (Wendt 1962). Genetic counselling, he argued to TV audiences, was one countermeasure against the predicted increase of ‘hereditary diseases’ (Wendt, n.d., quoted from Bock and Biermann 2017, min 03:16–03:37).39 In the meantime, at FDC meetings, this argument developed into an economic reasoning for primary prevention of disability by counselling, instead of support structures. ‘Even if it is an unpopular goal’, Nitsch and FDC treasurer Karl M Döll, a bank attorney, concluded in an internal meeting, ‘it is better to prevent hardship, than to alleviate it.”40 FDC members, such as Horst Bickel, a Heidelberg paediatrician, took this argument into political discourse. Bickel et al calculated the costs saved by counselling, amniocentesis and abortion, compared with the cost for the care of people with Down syndrome, for scientific and political audiences (Bickel, H. et al 1977).41 This argument was repeated by government representatives to ultimately justify the funding decisions for the aforementioned West German polite project on human genetic counselling (Bundesministerium für Jugend and Familie und Gesundheit 1979, 7).

Productivity was a major category in this debate. ‘Hereditary diseases’, Wendt stated to potential funders, also mean a ‘loss of productivity’ (Wendt 1970, 156). This is why the counselling environment framed by *Our Child Shall Be Healthy*, as he argued elsewhere, targete not only ‘tainted’ families but also those with a ‘social incapability’ (quoted after Reyer 2003, 183). After the oil crisis (1973), these arguments gained new popularity. In this period, West German nation building, as scholars noted, relied on transformations in the scientific landscape (Jasanoff 2007, 7), as well as it was shaped by the economic boom of the mid-1950s. At the dawn of the ‘golden age’ of Western capitalism, industrial growth represented stability and prosperity (Uekötter 2015, 81). Chemical and pharmaceutical companies such as Bayer AG or Merck AG, following the world leader IG Farben after 1945, ruled international markets and national research and development (R&D) (Bernsee 2020). R&D departments and drug consumption grew. Bayer’s introduction of the first European contraceptive pill (1961) ignited negotiations over uncertain gendered risks and the female body, amidst weak regulatory politics and contested medico-scientific expertise (Kessel 2009; Silies 2013). Against the backdrop of the routes that were taken in (pharma)industrial production on the one hand, and in reproductive counselling in the 1960s and 1970s, such as at university departments, on the other hand, the FDC’s economic–liberal position stuck out. According to confidential correspondence of federal ministry, the FDC’s focus on productivity and prosperity, instead of charity, was one reason, why, by the early 1970s, the Christian-conservative ministry withdrew funding.42 However, only by the early 1990s, with the rise of the disability rights movement, the paths taken in reproductive health counselling with *Our Child Shall Be Healthy* were challenged.

## Conclusion: communication and decision making

This paper reconstructed a leaflet as a communicative and paper technology in the post-1945, Western history of reproductive health. Scholars in history and the social sciences have argued that communication has structured how people do and do not reproduce (Hopwood et al. 2015). Going beyond the succession of technologies which medicine has used, the complex work of communication is built around the social and material worlds of leaflets, as well as printed books, journals, magazines, figurines, comics, exhibitions, film, radio, television and the internet. If used as communicative technologies or as part of practices of communication, these media are central to histories of reproduction. Drawing on the archives of a Marburg-based charity, today located in Berlin and building a cornerstone of the city’s infrastructure in reproductive healthcare, this paper tracked the uses, the production and dissemination of one of the most popular counselling leaflets in West Germany, named *Our Child Shall Be Healthy*. It thereby aims to contribute to a reconstruction of communication in counselling encounters between different actors, including industrialists, couples, bank attorneys, scientists and politicians, in a transformative period in the aftermath of thalidomide.

The West German context is known for approaches in genetic and reproductive health counselling, after 1945, deeply rooted in explanatory standards established under Nazi dictatorship (Klee 2001; Weingart 1988, 274). Only from the 1980s, these approaches were replaced by non-directive, participatory, non-coercive models (Cottebrune 2015; Waldschmidt 1996, 210). However, while the clear legacies of eugenics and racial hygienics are well researched, other details of this transformative period, such as the paper technologies that formed communication in counselling encounters, concepts, or actors and their entanglements, have not been examined in detail.

What factors explain the new way of communication in counselling interactions, by the late 1970s, including analogies of a choice of the best apple to the conscious choice of child? One answer is the integration of institutions, experts, stakes and social contexts into the making of a powerful counselling leaflet that formed counselling encounters at this moment in German history. The communication, by the *Our Child Shall Be Healthy*, of beneficial individual choices and the framing of reproductive decisions, as analogous to decisions on a market, as I argue, reflects significant entanglements between these different actors and their concepts of reproductive health after the boom (Doering-Manteuffel and Raphael 2008). These entanglements that informed the making of *Our Child Shall Be Healthy* help to understand how a combination of economic reasoning with an individual medical and preventive approach became a new guideline. A detailed study of the popular leaflet, in the context of its predecessors and audience critiques, also allows tracking of the developments contributing to what was often perceived as an ambivalence in the history in reproduction in West Germany: a larger shift in perspective in the debate on reproductive health—from society named as beneficiaries of certain interventions to the individual—and at the same time a persistence of arguments such as social and economic burden. In *Our Child Shall Be Healthy*, these arguments were combined with an implicit equation of higher social class and emotional well-being, as well as a rather directive approach to counselling encounters that leaves the final decision to the counselled.

The counselling leaflet reflects one of several tools for communicating specific and sometimes competing concepts of communicating reproductive health and risks, and related practices, emotions and interactions. Moreover, as this example showed, concepts travelled between counselling leaflets, student textbooks, popular lotteries, or advertisement campaigns at gas stations and in schools as a whole cosmos of communicative and paper technologies forming the histories of reproductive health that this paper has only started to recover.

## Data Availability

Data sharing not applicable as no datasets generated and/or analysed for this study.

## Notes

1. Stiftung für das behinderte Kind (n.d.). Unser Kind soll gesund sein (ca 1979–1980). Archive of the FDC, Berlin Charité, Campus Virchow.

2. Adoption is not a topic in West German reproductive Counselling at that time. I thank Heather Dron for pointing me to this question.

3. German orig.: Genetische Familienberatung. Ein Handbuch für Studenten und Ärzte.

4. Stiftung für das behinderte Kind (1973). Protokollnotiz über die Besprechung im Bundesministerium für Jugend, Familie und Gesundheit am 13.4.1973. 3. Bundesarchiv Koblenz/B-189/ 28123: Stiftung für das behinderte Kind.

5. Innenministerium Hessen (1967). Stiftungsurkunde ”Stiftung für das behinderte Kind”, 15.3.1967. Archive of the FDC, Berlin Charité, Campus Virchow, Beschlüsse, Verfassung, Aufsicht.

6. Stiftung für das behinderte Kind (1967). Erster Bericht an die Kuratoren der Stiftung für das behinderte Kind für den Zeitraum 12.6.1967 bis 30.11.1967. 10. Archive of the FDC, Berlin Charité, Campus Virchow, Kuratorium, Protokolle und Rundbriefe.

7. Stiftung für das behinderte Kind (1967). Erster Bericht an die Kuratoren der Stiftung für das behinderte Kind für den Zeitraum 12.6.1967 bis 30.11.1967. 1. Archive of the FDC, Berlin Charité, Campus Virchow, Kuratorium, Protokolle und Rundbriefe.

8. Strobel, K. (n.d.). Auszug aus Rede bei der konstituierenden Sitzung der Stiftung in Bad Godesberg, 12.6.1967. In: Stiftung für das behinderte Kind. Informationsheft über die Stiftung für das behinderte Kind. 2. Archive of the FDC, Berlin Charité, Campus Virchow, Kuratorium, Protokolle und Rundbriefe 67-82.

9. Stiftung für das behinderte Kind (1967, 1974, 1991). Satzung. §2. Archive of the FDC, Berlin Charité, Campus Virchow, Beschlüsse, Verfassung, Aufsicht, Jahresabschlüsse 94-99.

10. Stiftung für das behinderte Kind (1968). Protokoll der Vorstandssitzung, 6.7.68. 2. Archive of the FDC, Berlin Charité, Campus Virchow, Vorstand Protokolle 1967-88-2; Stiftung für das behinderte Kind (1974). Protokoll der Kuratoriumssitzung, 4.4.1974. 4. Archive of the FDC, Berlin Charité, Campus Virchow, Kuratorium, Protokolle, Rundbriefe 1967-82.

11. Stiftung für das behinderte Kind (1971). Protokoll Vorstandssitzung, 16.10.1971. 4. Archive of the FDC, Berlin Charité, Campus Virchow, Vorstand Protokolle 1967-88-2.

12. Stiftung für das behinderte Kind (1975). Protokoll Vorstandssitzung, 21.08.1975. 2. Archive of the FDC, Berlin Charité, Campus Virchow, Vorstand Protokolle 1967-88-2.

13. Stiftung für das behinderte Kind (1975). Protokoll der Vorstandssitzung, 21.03.1975. 2. Archive of the FDC, Berlin Charité, Campus Virchow, Vorstand Protokolle 1967-88-2.

14. Stiftung für das behinderte Kind (1975). Protokoll der Vorstandssitzung, 20.06.1975. 4. Archive of the FDC, Berlin Charité, Campus Virchow, Vorstand Protokolle 1967-88-2.

15. Stiftung für das behinderte Kind (1975). Protokoll Vorstandssitzung, 21.08.1975. 2. Archive of the FDC, Berlin Charité, Campus Virchow, Vorstand Protokolle 1967-88-2.

16. Stiftung für das behinderte Kind (1975). Protokoll der Vorstandssitzung, 21.03.1975. 2. Archive of the FDC, Berlin Charité, Campus Virchow, Vorstand Protokolle 1967-88-2.

17. Stiftung für das behinderte Kind (1975). Protokoll der Vorstandssitzung, 20.06.1975. 4. Archive of the FDC, Berlin Charité, Campus Virchow, Vorstand Protokolle 1967-88-2.

18. Stiftung für das behinderte Kind (1975). Protokoll Vorstandssitzung, 21.11.1975. 3. Archive of the FDC, Berlin Charité, Campus Virchow, Vorstand Protokolle 1967-88-2.

19. Stiftung für das behinderte Kind (1976). Protokoll der Vorstandssitzung, 11.8.1976. 3. Archive of the FDC, Berlin Charité, Campus Virchow, Vorstand Protokolle 1967-88-2.

20. Stiftung für das behinderte Kind (1976). Protokoll der Vorstandssitzung, 11.8.1976. 3. Archive of the FDC, Berlin Charité, Campus Virchow, Vorstand Protokolle 1967-88-2.

21. Stiftung für das behinderte Kind (1977). Protokoll Vorstandssitzung, 16.2.1977. 3–4. Archive of the FDC, Berlin Charité, Campus Virchow, Vorstand Protokolle 1967-88-2.

22. Stiftung für das behinderte Kind (1979). Protokoll Kuratoriumssitzung, 15.4.1979. 2. Archive of the FDC, Berlin Charité, Campus Virchow, Kuratorium, Protokolle und Rundbriefe 67-82.

23. Stiftung für das behinderte Kind (1978). Protokoll der Vorstandssitzung, 9.11.1978. 3. Archive of the FDC, Berlin Charité, Campus Virchow, Vorstand Protokolle 1967-88-2.

24. Stiftung für das behinderte Kind (1978). Protokoll der Vorstandssitzung, 9.11.1978. 3. Archive of the FDC, Berlin Charité, Campus Virchow, Vorstand Protokolle 1967-88-2.

25. Stiftung für das behinderte Kind (n.d.). Bericht des Vorstandes an die Mitglieder des Kuratoriums. 2. Archive of the FDC, Berlin Charité, Campus Virchow, Kuratorium, Protokolle und Rundbriefe 67-82.

26. Stiftung für das behinderte Kind (n.d.). Bericht des Vorstandes an die Mitglieder des Kuratoriums. 2. Archive of the FDC, Berlin Charité, Campus Virchow, Kuratorium, Protokolle und Rundbriefe 67-82.

27. Stiftung für das behinderte Kind (1978). Protokoll der Vorstandssitzung, 9.11.1978. 5. Archive of the FDC, Berlin Charité, Campus Virchow, Vorstand Protokolle 1967-88-2.

28. Stiftung für das behinderte Kind (n.d.). Bericht des Vorstandes an die Mitglieder des Kuratoriums. 2. Archive of the FDC, Berlin Charité, Campus Virchow, Kuratorium, Protokolle und Rundbriefe 67-82.

29. Stiftung für das behinderte Kind (1979). Protokoll der Vorstandssitzung, 19.10.1979. 1. Archive of the FDC, Berlin Charité, Campus Virchow, Vorstand Protokolle 1967-88-2.

30. Stiftung für das behinderte Kind (1988). Protokoll Vorstandssitzung, 30.5.1988. 3. Archive of the FDC, Berlin Charité, Campus Virchow, Vorstand Protokolle 1967-88-1.

31. Stiftung für das behinderte Kind (1980). Protokoll der gemeinsamen Sitzung von Kuratorium und Vorstand, 24.10.1980. 2. Archive of the FDC, Berlin Charité, Campus Virchow, Kuratorium, Protokolle und Rundbriefe 67-82; Stiftung für das behinderte Kind (1982). Vorstandsprotokoll, 28.6.1982. 5. Archive of the FDC, Berlin Charité, Campus Virchow, Vorstand Protokolle 1967-88-2.

32. Stiftung für das behinderte Kind (1986). Bericht des Vorstandes an die Mitglieder des Kuratoriums, 25.3.1986. 4. Archive of the FDC, Berlin Charité, Campus Virchow, Vorstand Protokolle 1967-88-1.

33. Stiftung für das behinderte Kind (1988). Protokoll Vorstandssitzung, 30.5.1988. 3. Archive of the FDC, Berlin Charité, Campus Virchow, Vorstand Protokolle 1967-88-1.

34. Stiftung für das behinderte Kind (n.d.). Mitglieder des Kuratoriums. Bundesarchiv Koblenz/ B-189/ 28123: Stiftung für das behinderte Kind.

35. Stiftung für das behinderte Kind (1972). Protokoll der Vorstandssitzung, 10.3.1972. Bundesarchiv Koblenz/B 189/ 28123: Stiftung für das behinderte Kind.

36. Stiftung für das behinderte Kind (1972). Protokoll der Vorstandssitzung, 10.03.1972. 4–5. Archive of the FDC, Berlin Charité, Campus Virchow, Vorstand Protokolle 1967-88-2.

37. Stiftung für das behinderte Kind (1967). Ergebnisprotokoll, 2.11.1967. 4. Archive of the FDC, Berlin Charité, Campus Virchow, Vorstand Protokolle 1967-88-2; Stiftung für das behinderte Kind (1968). Protokoll über die Vorstandssitzung, 31.5./1.6.1968. 3. Archive of the FDC, Berlin Charité, Campus Virchow, Vorstand Protokolle 1967-88-2; Stiftung für das behinderte Kind (1980). Protokoll der Vorstandssitzung, 8.10.1980. 3. Archive of the FDC, Berlin Charité, Campus Virchow, Vorstand Protokolle 1967-88-2.

38. Stiftung für das behinderte Kind (1968). Mietvertrag für gewerbliche Räume zwischen Farbwerke Hoechst und Stiftung für das behinderte Kind. Archive of the FDC, Berlin Charité, Campus Virchow, Verwaltung 1967-72.

39. German Orig.: Genetische Beratung kann erreichen, dass Erbkrankheiten nicht allzu sehr an Häufigkeit zunehmen. […] Das Hauptziel der genetischen Beratung aber ist, dass die heute gezeugten und geborenen Kinder gesund sein sollten.

40. Nitsch, K. (n.d.). Entwurf Brief an die Kuratoren der Stiftung für das behinderte Kind. 2. Archive of the FDC, Berlin Charité, Campus Virchow, Vorstand Protokolle 1967-88-2.

41. Stiftung für das behinderte Kind (1966). Wissenschaftliche Basis des Hilfsprogramms, 1.6.1966, Marburg/Lahn. Bundesarchiv Koblenz/ B-189/ 28123: Stiftung für das behinderte Kind.

42. Stiftung für das behinderte Kind (1973). Protokoll der Vorstandssitzung, 12.11.1973. Bundesarchiv Koblenz/ B-189/ 28123: Stiftung für das behinderte Kind.
